# Permafrost preservation reveals proteomic evidence for yak milk consumption in the 13^th^ century

**DOI:** 10.1038/s42003-023-04723-3

**Published:** 2023-03-31

**Authors:** Alicia R. Ventresca Miller, Shevan Wilkin, Jamsranjav Bayarsaikhan, Abigail Ramsøe, Julia Clark, Batsuren Byambadorj, Sandra Vanderwarf, Nils Vanwezer, Ashleigh Haruda, Ricardo Fernandes, Bryan Miller, Nicole Boivin

**Affiliations:** 1 Department of Anthropology, University of Michigan, Ann Arbor, 48109 MI USA; 2 Museum of Anthropological Archaeology, University of Michigan, Ann Arbor, 48109 MI USA; 3 Department of Archaeology, Max Planck Institute for the Science of Human History, Kahlaische Strasse 10, 07745 Jena, Germany; 4 Institute for Evolutionary Medicine, Faculty of Medicine, University of Zürich, 8057 Zürich, Switzerland; 5 Australian Research Centre for Human Evolution (ARCHE), Griffith University, Brisbane, 4111 QLD Australia; 6 National Museum of Mongolia, Juulchin Street-1, Ulaanbaatar, Mongolia; 7 Section for GeoGenetics, The GLOBE Institute, University of Copenhagen, Copenhagen, Denmark; 8 NOMAD Science, Glen, MT USA; 9 Flinders University: Department of Archaeology, Flinders University, Bedford Park, 5042 Adelaide, SA Australia; 10 Department of Sociology, Social Work and Anthropology, Utah State University, Logan, UT USA; 11 Department of Anthropology and Archaeology, National University of Mongolia, Baga toiruu-44, Ulaanbaatar, 46a Mongolia; 12 School of Archaeology, University of Oxford, 1 South Parks Road, Oxford, UK; 13 Department of Archaeology, University of Exeter, Laver Building, North Parks Road, Exeter, UK; 14 Faculty of Arts, Masaryk University, Arne Nováka 1, 602 00 Brno-střed, Czechia; 15 History of Art Department University of Michigan, Ann Arbor, 48109 MI USA; 16 School of Social Science, University of Queensland, Brisbane, QLD Australia; 17 Department of Archaeology, University of Calgary, Calgary, AB Canada; 18 Smithsonian Institution, New York, NY USA

**Keywords:** Peptides, Palaeoecology

## Abstract

Domesticated yaks endure as iconic symbols of high-altitude frozen landscapes, where herding communities depend on their high-fat milk, transport, dung, and natural fibers. While there is established proteomic evidence for ancient consumption of ruminant and horse milk in the mountains and steppes of northern Eurasia, yak dairy products have yet to be detected. Yak domestication and the species’ dispersal from Tibet into the mountainous zones to the north are also poorly resolved due to a paucity of zooarchaeological data. To examine the potential of paleoproteomics to shed light on domesticated yak in Mongolia, we analyzed human dental calculus from Mongol era elite individuals recovered from permafrost burials in Khovsgol province, where people continue to herd yak to this day. We report the first evidence for yak dairy consumption, linked to local resource control. In addition, we confirm a large diversity of recovered whey, curd, tissue, and blood proteins, likely reflecting the excellent preservation conditions found at permafrost sites.

## Abstract (web summary)

Proteomic evidence from permafrost human dental calculus indicates consumption of yak milk in Mongolia as early as 1270 CE.

## Introduction

The domesticated yak (*Bos grunniens*)^1^ is well adapted to cold and high-altitude alpine tundra ecosystems. Yaks are able to survive temperatures lower than −40 °C, as well as obtain water by eating snow and ice, while accessing covered winter forage, including grasses, shrubs, moss, and lichens, by digging under snow^2,3^. For communities living at high altitudes in eastern Eurasia, yak products provide an important source of calories, as well as commodities for local consumption and exchange. Yak milk is high in fat, making it ideal for producing butter and cheese, and for the manufacture of candles and lamps^4^. The hair from yaks can be made into textiles and felts^5,6^, while the animal’s dried dung functions as an important fuel source for heating and cooking, making it a critical resource in regions where wood is scarce^4^. Yaks are regularly used for traction and transport, connecting human communities at high altitudes.

**Fig. 1:**
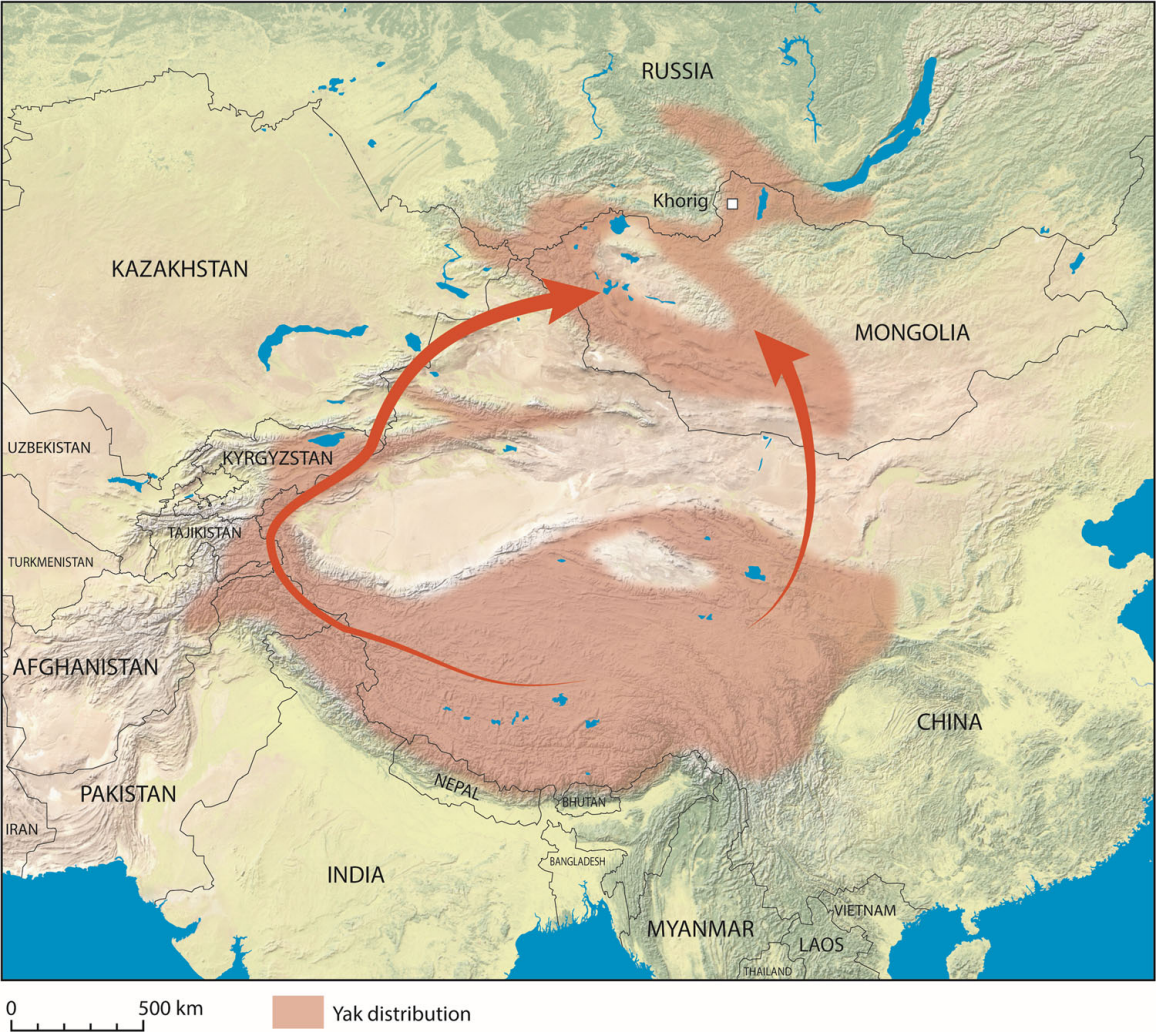
Distribution of the yak across Asia during historical and modern periods. Reconfigured from ref. ^70^. Arrows suggest hypothesized pathways for the spread of domesticated yak from northern Tibet into Mongolia. This map was produced using Adobe Illustrator CC 2020 and using the Natural Early Data maps from https://www.naturalearthdta.com/downloads/ by AVM and Michelle O’Reilly (Graphic Designer for the MPI-SHH) and John Klausmeyer (Illustrator for the UMMAA).

Yaks have accordingly been central to human settlement of the high-altitude regions of eastern Eurasia, notably the Tibetan Plateau and the mountains of Mongolia. In the harsh environments of the Tibetan Plateau, yaks provide primary sources of food, heat, and transport for rural communities, while in northern Mongolia, yak products diversify human diets and provide commodities for exchange^2^. The human-mediated dispersal of early yaks from their domestication center in Tibet remains poorly understood, in significant part due to a paucity of zooarchaeological evidence^7,8^. The center of yak domestication has been broadly placed in the Tibetan plateau at ~3000 BCE, with osteological evidence at the site of Qugong (~1750-1100 BCE)^9^. A number of early routes of expansion beyond the Tibetan plateau have been proposed^10,11^ (Fig. 1). The best-supported routes of dispersal into Mongolia are from the eastern Tibetan plateau to the west towards the Pamir Mountains or north towards the Altai and Khangai Mountains in Mongolia^11,12^.

Mongolia has, to date, yielded little evidence of the yak’s early history in the region, and the domesticated status of yaks identified is often unclear. The only identifiable archeological specimen recovered is a single yak cranium, from the site of Denjiin Navtan, preliminarily dated to the Late Bronze or Early Iron Age (~1000 to 500 BCE)^13,14^. Beyond this, rock art thought to be of Bronze Age antiquity has been interpreted as representing yak caravans^15^ (Fig. S4). Preliminary zooarchaeological reports from Mongol-era sites have not revealed any evidence of yaks^16^. Nonetheless, archaeological and historical records demonstrate that yak products, including yak hair felts^17^, had become an important part of the steppe political economy by the Xiongnu era (200 BCE to 200 CE). In a Xiongnu royal tomb in central Mongolia, archeologists recovered a bag with hair from humans, horses, and yaks^18^, while remains of a wild yak were recovered from the Xiongnu site of Ivolga in Buryatia^19^. Yaks also appear on prestigious Xiongnu belt buckles and horse gear (phallera), suggesting they were at least symbolically prominent in the empire, especially in the mountainous west^20,21^. Han Chinese texts from the same period refer to the political importance of yak goods, and clarify that yak products were among tribute goods coming from the western areas (Xinjiang, Gansu)^22,23^. Approximately 1000 years later, Mongol-era textual references to the yak are sparse, but indicate that yak hair products continued to be important components of tribute and were used in infantry standards and other elite regalia^24,25^. While diverse yak product uses are evident in historical texts, mentions of domesticated yaks, yak dairying, or yak milk consumption are lacking.

**Fig. 2:**
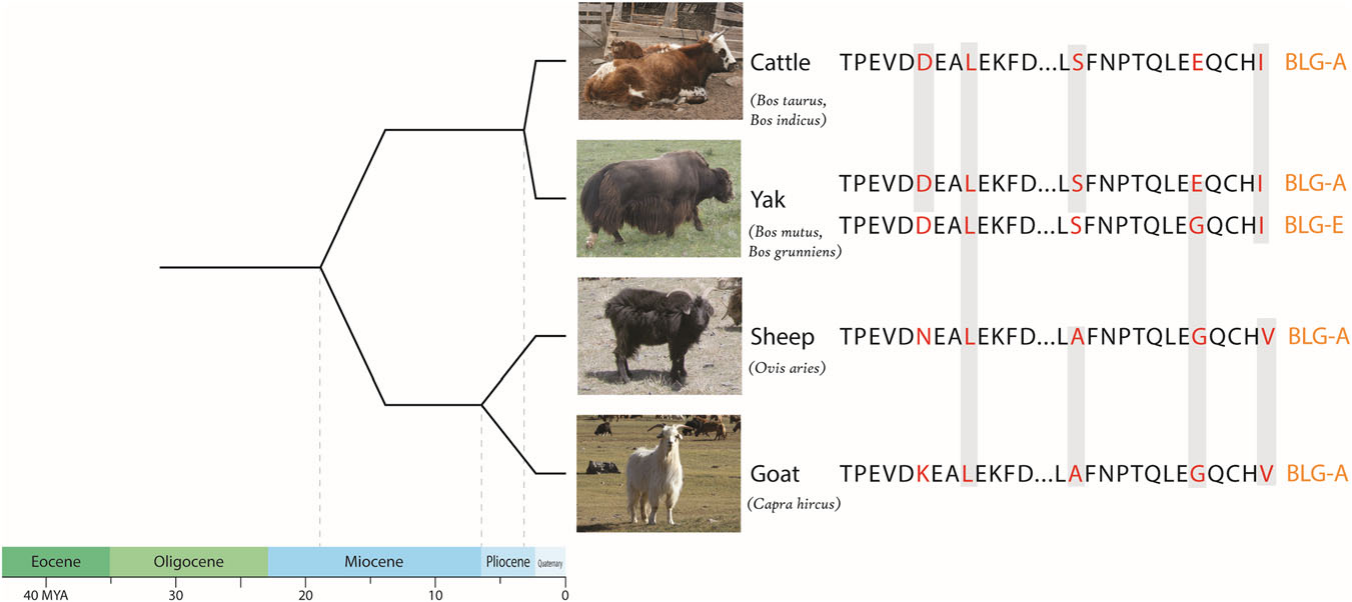
Alignment of two tryptic BLG peptides that allow for species differentiation. The second yak peptide represents the last peptide (14 amino acids), which allows for the identification of *Bos grunniens*/*mutus* (BLG-E) (36). Here we compare the sequences from several key dairy animals (cattle, yak, sheep, and goat) to demonstrate the increasing sequence differences as species diverge phylogenetically. Accession codes in UniProt for *BLG A* is P02754 (shown in UniProt as Bos taurus), and *BLG E* is L8J1Z0 (shown in UniProt as *Bos mutus*).

Given the paucity of archaeological and historical data for domesticated yak in Mongolia, proteomic analysis offers significant potential to shed light on the species’ spread and early uses. The proteomic analysis of ancient dental calculus supports studies of dietary sources, especially milk consumption in the past^26–35^. To date, evidence for yak milk has not been recovered in paleoproteomic studies at any site in Mongolia or Eurasia, more broadly^30,36–38^. Previous analyses of dental calculus from the Mongol-era focused on individuals from primarily non-elite contexts, all of whom lacked evidence for yak dairy consumption^30^. While peptide sequences for the most commonly recovered ancient milk protein, beta-lactoglobulin (BLG), often enable taxonomic identifications to cattle, sheep, goats, and horses, the yak BLG sequence is less straightforward^39^. Yaks (both domesticated *Bos grunniens* and wild *Bos mutus*) share over 99% of the sequence for beta-lactoglobulin with other *Bos* species, including *Bos taurus* and *Bos indicus*. Both domestic and wild yaks produce two paralogous versions of BLG; one (BLG-A) is identical to the *Bos taurus* sequence, and thus taxonomically less useful, while the other (BLG-E) differs from the *Bos taurus* sequence (BLG-A) by a single amino acid in the final tryptic peptide of the protein sequence^39^ (Fig. 2). This means that the identification of yak-specific peptides from archaeological dental calculus is more challenging than for other species, due to the reliance on the recovery of the final tryptic peptide of BLG-E. To date, when the BLG peptide has been recovered, it has yielded only the *Bos taurus*- and caprine-specific versions of the sequence^30,36,37^.

**Fig. 3:**
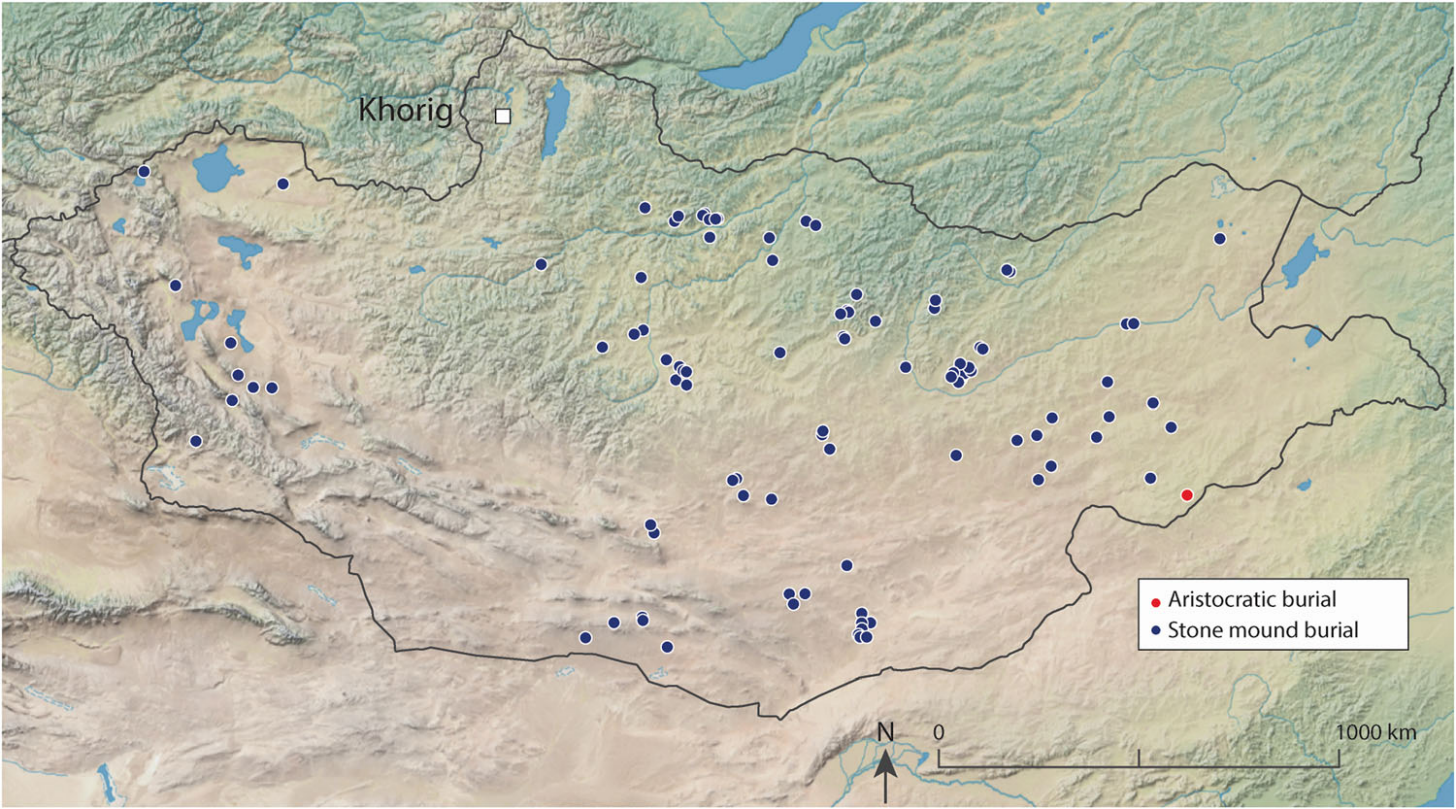
Location of the elite cemeteries of Khorig I and Khorig II (identified here as Khorig) in relation to other Mongol-era burials (elite and non-elite) in Mongolia. This map was produced using Adobe Illustrator CC 2020 and using the Natural Early Data maps from https://www.naturalearthdta.com/downloads/ by AVM and Michelle O’Reilly (Graphic Designer for the MPI-SHH) and John Klausmeyer (Illustrator for the UMMAA).

In order to recover archaeological yak milk peptides, and to examine past yak milk consumption, we sought to target a site with well-preserved remains and good potential for biomolecular preservation. We accordingly analyzed ancient proteins preserved in dental calculus from human burials at the site of Khorig on the northern periphery of the Mongol Empire (Khovsgol province)^40,41^ (Figs. 1, 3). Elite burials at the Khorig cemeteries are situated within the permafrost along high-altitude ridgelines in the Khovsgol mountains (Khoridol Saridag mountains) of northern Mongolia, permitting exceptional preservation of organic remains, including silk, felt, and leather, as well as residues in ceramic vessels^40,41^. At the base of the burial pits, we often encountered ice or frozen earth. The cemeteries of Khorig I and II span a period beginning just before the unification of the Mongol Empire (1206 CE) and through the Yuan Dynasty period (1271–1368 CE)^42,43^. Lavish grave goods indicate that many of the individuals buried in the cemeteries were elite members of society, with only one other aristocratic cemetery identified for this period in Mongolia (Fig. 3). The Khorig site is at present being actively looted and was thus investigated as part of a salvage excavation conducted in 2018 and 2019^42,43^.

## Results

Proteins were extracted from the dental calculus of 11 individuals from Khorig. We assessed samples using a custom-made oral signature screening database (OSSD)^44^, since well-preserved ancient dental calculus samples should contain an authentic oral signature (Fig. S1). Protein recovery across the samples was variable, but 10 of 11 samples yielded proteins typically found in the oral cavity (Table S1). The total number of proteins identified per calculus sample was similar to, or greater than, that which has been reported in other samples from Central and Inner Asia^30,36^.

**Fig. 4:**
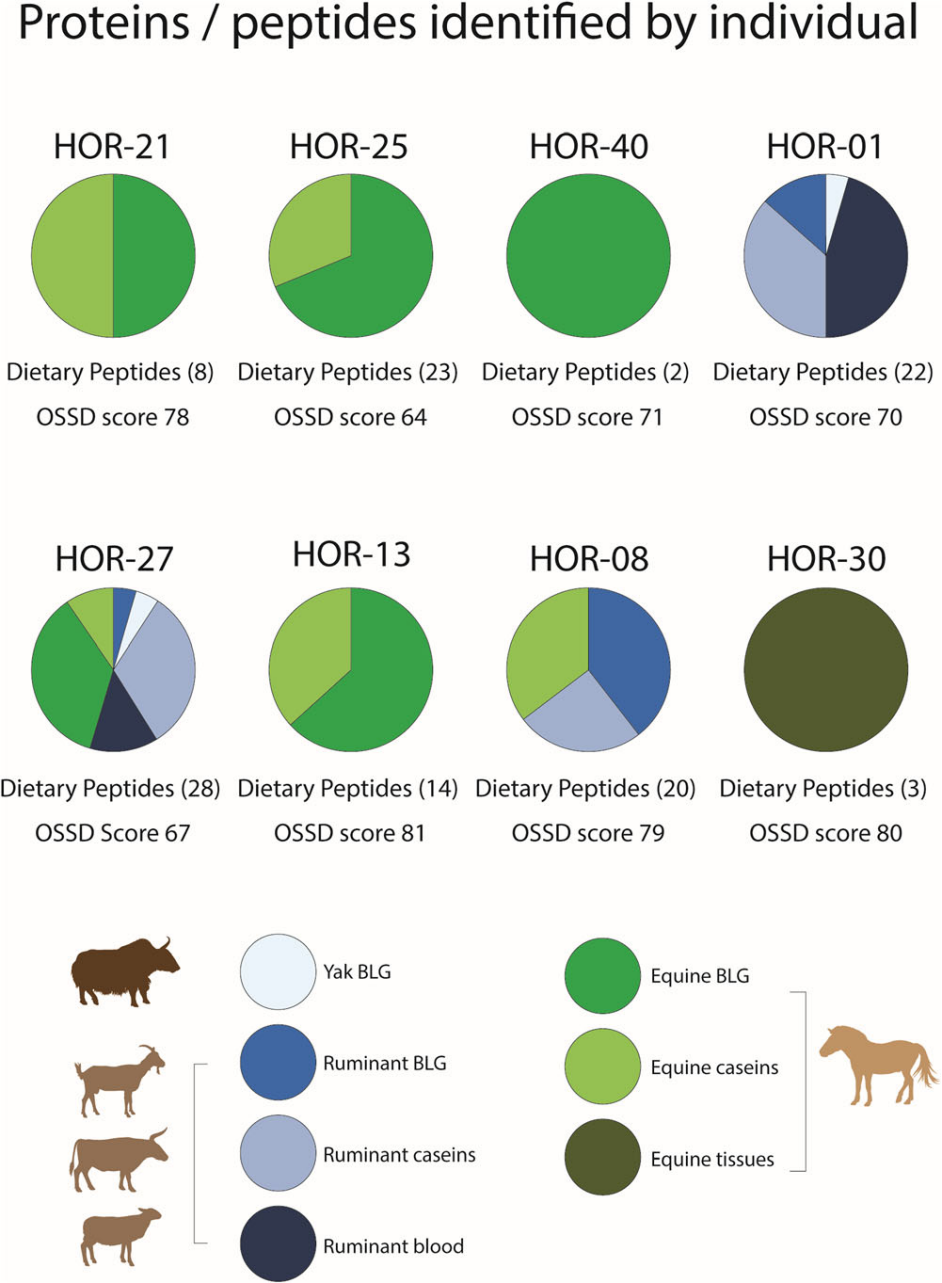
Proportion of proteins/peptides for each individual. Each individual had varying proportions of dietary peptides found in their dental calculus, which was related to type of food and species of animal consumed.

**Fig. 5:**
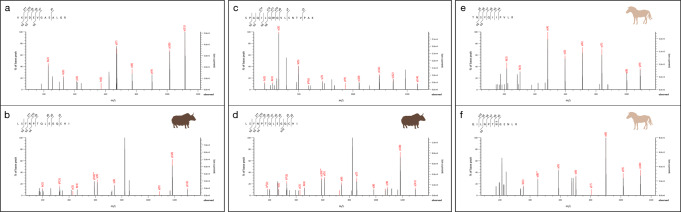
Spectra for a range of peptides identified in dental calculus at Khorig. **a** Hemoglobin subunit beta specific to Pecora (HOR-01). **b** BLG-E specific to *Bos grunniens*/*mutus* (HOR-01). **c** Kappa casein specific to Bovinae (HOR-27). **d** BLG-E specific to *Bos grunniens*/*mutus* (HOR-27). **e** Alpha-S2 casein specific to *Equus* (HOR-08). **f** Beta-casein specific to *Equus* (HOR-08).

Of the dental calculus samples that passed our preservation assessment, eight of ten individuals (80%) showed evidence of consumption of animal products (milk, meat, blood) (Fig. 4). The presence of specific peptide sequences enabled the identification of genus- or species-specific peptides for four taxa: *Equus* (horse, donkey), *Ovis* (sheep), *Bos* (cattle), and *Bos grunniens/mutus* (yak), with other peptide spectral matches (PSMs) fitting into broader taxonomic classifications such as Caprinae, Bovinae, Bovidae, and Pecora (all even-toed ruminants) (see Fig. 5 and Supplementary Note. 1). Peptides specific to milk proteins from ruminant species include beta-lactoglobulin (BLG, including yak variant BLG-E), beta-casein, alpha-S1-casein, alpha-S2-casein, and kappa casein. From horses, the primary milk protein recovered was BLG1 (Supplementary Note. 1), while Lysozyme C (milk isozyme), alpha-lactalbumin, alpha-S2-casein, and beta-casein were also recovered. Seven out of the eight dental calculus samples that contained evidence of dairy consumption also yielded horse milk peptides. Yak-specific milk peptides were identified in calculus samples from two individuals. Detailed results, including post-translational modifications, Mascot ion scores, and e-values, as well as peptide and protein false discovery rates (FDR), are shown in Table S2. We also provide spectra for a range of peptides from the dental calculus of three individuals (Fig. 5).

The results of this study include evidence for the consumption of yak-specific (*Bos grunniens/mutus*) milk in two of the individuals analyzed, with calibrated radiocarbon date ranges from 1170 to 1270 CE (HOR-27) and from 1287 to 1397 CE (HOR-01). Dental calculus from the earlier individual (HOR-27) yielded eight different milk proteins (BLG; BLG1; alpha-lactalbumin; Lysozyme C, milk isozyme; beta-casein; alpha-S1-casein; alpha-S2-casein; kappa casein). Sequence data show that these milk proteins included 23 peptides useful for taxonomic identifications. Specific matches were possible to *Bos mutus* species (*n* = 1 peptide), as well as to higher order groupings like the genus *Equus* (*n* = 12), the subfamily Bovinae (*n* = 2), the Bovidae family (*n* = 4), and the Pecora infraorder (*n* = 4). In addition, this individual may have consumed dairy from other species included in the subfamily Bovinae (cattle or cattle/yak hybrids) or the family Bovidae (cattle, sheep, and/or goat). This individual’s calculus also contained evidence for ruminant-specific blood proteins, indicating ruminant meat (or blood) consumption. Similarly, the calculus from a second individual (HOR-01) contained evidence for ruminant milk proteins with taxonomic classifications specific to *Bos grunniens/mutus* (*n* = 1), as well as broader level taxonomic groups—*Bos* (*n* = 1) (which includes *Bos taurus, Bos grunniens/mutus, Bos indicus*, and the hybrid of *Bos mutus/indicus*), Bovinae (*n* = 3), Bovidae (*n* = 2), and Pecora (*n* = 4) (all Artiodactyla). In the same individual, we also identified evidence for two ruminant-specific blood proteins.

A third individual (HOR-08) had calculus that contained peptide identifications for BLG and alpha-S1-casein that were taxonomically identified to *Ovis*, Caprinae (sheep or goat), and one taxonomically ambiguous peptide that could be matched to sequences for either Bovinae or *Ovis*. The sample additionally contained equine-specific protein identifications to beta-casein and alpha-S2-casein, which represents the first identification of caseins from horse milk in archaeological samples. The calibrated dates for this individual range from 1269 to 1388 CE (Supplementary Note. 1).

Several individuals (HOR-40, HOR-13, HOR-21, and HOR-25) had peptide spectral matches (PSMs) to equine milk proteins. This included BLG1, Lysozyme C (milk isozyme), and alpha-lactalbumin. Finally, one individual (HOR-30) lacked peptide matches specific to milk proteins, but did contain three PSMs to *Equus* (horse) serum albumin, which is a protein found in tissues (e.g., blood, fat, sweat, or urine).

## Discussion

Our results suggest that yak milk was likely consumed in Mongolia by ~1270 CE. Given the paucity of existing evidence for yak dairying and, indeed, yak exploitation more broadly in the Mongolian and greater global archaeological record, this finding is significant. We acknowledge that this is a relatively late date for yak milk consumption, which probably occurred in earlier eras but has until now not been identified. Our study highlights the potential of paleoproteomics to shed light on yak management, by detecting yak dairy proteins in dental calculus and allowing for inferences to be made regarding the milking of yaks during the Mongol period. Our findings provide a useful complement to textual evidence indicating the yak’s symbolic importance in Mongolia in the historical period, suggesting that its use may have been more widespread than formerly appreciated. Nonetheless, yaks were only one of several different species whose milk was consumed by the elite population buried at the Khorig cemeteries. Dairy products from horses, as well as cattle, sheep, and/or goat were consumed perhaps more regularly than yak milk, although estimates of proportionality are not possible in the present study.

During the Mongol Empire (1206–1368 CE), economic trade flourished and there was a globalized exchange of animals, textiles, and spices across Eurasia and beyond^25,45,46^. At the northern frontier of the empire, located in modern-day northern Mongolia and southern Russia, elites may have controlled locally produced commodities that were unavailable in other regions^47^. In the high-altitude locales where the Khorig sites are located (Fig. 3), cattle, yaks, and yak-cattle hybrids flourish and their high-fat butter, milk, and hair likely constituted precious trade goods in the Mongol-era. Other finds recovered from Khorig burial contexts included large vessels filled with butter or fat with wicks, suggesting they were lamps. Yak butter lamps are an important part of Tibetan Buddhist traditions (Lamaism)^48^, and may have been produced at sites like Khorig for export to other locales within the empire, supporting elite livelihoods. The recovery of a gold Buddha (Fig. S3) at the Khorig site suggests that Buddhism was an important part of religious life amongst Khorig elites, and it became the main religion of the Yuan dynasty (1271–1368 CE). Lamps made with yak butter were likely utilized locally, perhaps in emulation of religious practices in Tibet (but cf refs. ^42,43^).

The challenges of identifying yak milk, which as noted, differs by only a single tryptic peptide from the milk of other species within the genus *Bos*, has until now impeded the identification of this species in palaeoproteomic studies of ancient dairying. Proteomic studies of dental calculus from other prehistoric and contemporaneous societies in eastern Eurasia^30,36^ have not identified yak-specific milk proteins, nor has analysis of preserved residues^49,50^. Our study indicates that yak-specific milk peptides are recoverable from archaeological dental calculus and can inform researchers about dietary intake as well as yak-related dairying practices. Nonetheless, recovery of yak-specific sequences, unless through targeted methods, will continue to depend on fortuity and excellent preservation.

While the Khorig finds currently stand as the earliest evidence globally for the consumption of yak dairy, little can be said about the broader early use of yak milk given the taxonomic challenges involved in its identification. It is likely that earlier populations in Mongolia utilized yak products, such as dairy and meat, as suggested by sparse zooarchaeological remains^14^ and artistic renditions^20^ (Fig. S4). Consumption of yak dairy by elites in the mountainous western region of Mongolia highlights the importance of yak products in the Mongol period. This finding is further supported by textual evidence for the use of yak hair in standards, felts, and other accessories. As noted, communities on the frontier of the Mongol empire likely controlled these highly sought-after, locally produced commodities, overseeing their trade into Mongol cities from the western and northern regions of the Empire^51^.

The exceptional preservation of the Khorig samples also permitted the identification of blood and tissue proteins from ruminants and horses. Our analysis recovered hemoglobin proteins from the infraorder Pecora and family Bovidae, which includes mammals with ruminant digestion, such as cattle, sheep, and goats. Also recovered in a single individual were three peptides from serum albumin, an abundant protein that, in this case, showed peptides specific to horses. Serum albumin can be expressed in various tissues (blood, fat, sweat, or urine). However, it seems likely that the recovery of serum albumin or hemoglobin in calculus can be linked to human ingestion of meat, organs, fat, or blood of ruminants or horses, the latter of which were likely only slaughtered on ceremonial occasions in the past, as is the case in modern contexts^52^. Mentions of boiling cauldrons filled with meat, and the bleeding of camels for consumption, are present in texts from the Mongol-era^25^. The consumption of foods containing blood was not unusual and is supported by our identification of blood and tissue proteins preserved in the calculus of several Khorig individuals.

Along with other unique proteomic finds, we have also identified evidence for horse-specific caseins (alpha-S2 and beta-casein), which have not previously been recovered from ancient samples. Caseins form the largest proportion of proteins in raw milk, with casein amounts increasing in many types of processed dairy (cheese, yogurt, butter) after the removal of the liquid whey portion. Despite their ubiquity, caseins preserve less well over the long term than BLG proteins, which are more commonly recovered in archaeological studies^53^. Overall, our study yielded a greater diversity of milk proteins than previously recovered from ancient and historic human dental calculus (refs. ^30–38^ and ^44^), with the extraordinary preservation conditions of the permafrost at the Khorig site likely enabling long-term persistence of rarely recovered proteins.

This study highlights the capacity of permafrost sites to yield uniquely preserved and informative samples. As with other biomolecules^54,55^, the preservation of proteins encapsulated in dental calculus is enhanced in cold environments^26^. The Khorig site is located in the Khovsgol mountains at elevations between 2000 and 2100 m, with continuous permafrost found on slopes at elevations over 2000 m^56^. The extent, continuity, and thickness of permafrost is massively impacted by climate change^56^, with major implications not only for ecosystems, but also for cultural heritage. The looting of the Khorig cemeteries is a direct result of its exposure due to melting permafrost, which not only left the site vulnerable to looting, but also initiated processes of biomolecular degradation of the site’s exceptionally well-preserved residues and artefacts. Our unique findings of the first yak milk, ruminant blood proteins, and horse caseins, through palaeoproteomic methods, highlight how climate change robs us of our future as well as some of the richest remains of our past. Heritage specialists must prioritize identification, recovery, conservation, and analysis of the rich artifactual record melting out of the permafrost in Mongolia and other northern regions of the globe^57,58^.

## Methods

Dental calculus was collected from 11 adult individuals from the Khorig I and II cemeteries. Samples were taken from human remains excavated in 2018 and 2019, the former curated at the National Museum of Mongolia in Ulaanbaatar and the latter collected directly after excavation in an on-site laboratory. Dental calculus was removed from each tooth using sterilized dental scalars and collected in 1.5 mL sterile Eppendorf tubes. Nitrile gloves and facemasks were used during sample collection to avoid contamination. Stored samples were transported to the Palaeoproteomics Laboratory at the Max Planck Institute for the Science of Human History in Jena, Germany. All materials from Mongolia were sampled and exported with permission from the National Museum of Mongolia.

The cemetery was initially dated through a material culture that included preserved silks, a Cizhou vessel, and a Buddha ornament (Fig. S3). The recovery of gold-woven textiles with distinctive motifs suggests that the individuals buried in the cemetery date to just before (~1115–1270 CE) and during the Yuan Dynasty period of the Mongol Empire (1271 to 1368 CE)^59^. In addition, three samples of human bone were sent to the SUERC Radiocarbon Laboratory in Glasgow for dating, yielding the following calibrated ranges (95.4%): 1287–1397 CE (SUERC-96725/HOR-01), 1269–1388 CE (SUERC-96726/HOR-08), and 1170–1270 CE (SUERC-96727/HOR-27) (for further discussion and methods see Supplementary Note. 1 and Table S3).

Calculus samples were demineralized and proteins were extracted using a single pot, solid phase sample preparation method (SP3) modified for dental calculus^60^. Both a positive control (archaeological sheep bone) and a negative control (extraction blank) were extracted along with each batch of dental calculus. Full details of the entire protocol for demineralization, extraction, and peptide clean-up are included at protocols.io under the 10.17504/protocols.io.bfgrjjv6. Proteins were extracted in a dedicated clean room within dead-air workstations. In an effort to decrease contamination, we wore clean room clothes, two pairs of solvent-resistant gloves, a face mask, safety glasses, a hair net, and clean room shoes. Following protein extraction, digested peptides were sent to the Functional Genomics Center Zurich for high-performance liquid chromatography–tandem mass spectrometry analysis (HPLC-MS/MS). The resulting raw MS/MS data were converted to Mascot generic files (mgf) using MSCovert from ProteoWizard (v.3.0.11781)^61^. These files were then searched against Swissprot and a custom-curated dairy protein database^30^ using Mascot MS/MS ion search engine (v.2.6.0)^62^. Mascot search settings were as follows: enzyme was set to “trypsin”; as we reduced and alkylated, the fixed modification was set for the carbamidomethylation of cysteine (C); we included variable modifications for deamidation of asparagine (N) and glutamine (Q) and oxidation of methionine (M); peptide mass tolerance was set at 10 ppm, and fragment mass tolerance was 0.01 Da; as we used a Q-Exactive orbitrap tandem mass spectrometer the instrument setting was chosen as ‘Q-Exactive’; and finally, we allowed for one monoisotopic mass shift.

PSM identifications via Mascot were filtered using an in-house developed tool, MS-MARGE (ref. ^63^ freely available at https://bitbucket.org/rwhagan/ms-marge/src/master/). PSMs with an e-value of above 0.01 and Protein ID’s with fewer than two unique PSMs were excluded. From these filtered data, PSM species identification matches were checked using NCBI BLASTp for specificity and uniqueness. For example, PSMs assigned to a species by Mascot may not be unique to a single species, and may match numerous species. BLASTp displays all matches to a specific amino acid sequence, and displays the least common taxonomic group by navigating to the “Taxonomy” section of the results. Dietary protein PSMs allocated to the genus Equus or infraorder Pecora and below are included in Table S2. All data files (raw, peak, and result files) for this study have been uploaded to the ProteomExchange (http://www.proteomexchange.org) under accession number: PXD024510 and project DOI: 10.6019/PXD024510.

### Preservation of ancient dental calculus

Research has shown that dental calculus traps proteins and peptides associated with the human oral proteome and oral microbiome^26,27,29,31–34,64^. However, the preservation of proteins within dental calculus samples can vary greatly across different regions and climates due to varying environmental contexts (temperature, aridity, groundwater, and curation). Here we use a previously tested method^44,60^ to assess the relative preservation and authenticity of the overall proteome of each calculus sample in our study. Dental calculus is produced when a dental plaque biofilm calcifies. Normally, this biofilm includes bacteria from the oral microbiome, and/or human immune response proteins produced in the mouth and salivary glands, and can contain evidence of proteins from dietary consumption that become trapped in its matrix.

Following a previous study^60^, we matched the identified peptide spectral matches (PSMs) for each sample for proteins included in the Oral Signature Screening Database (OSSD). Rather than running an independent search using the OSSD, we created a short script to search our results from Swissprot and our custom-created dairy database for common laboratory and environmental contaminants, human immune proteins found in the mouth, and entire proteomes of the most common oral microbiome bacteria. This way, the OSSD was only compared against PSMs that passed our data filtration step. For the full list of proteins and proteomes included, please see the previously published Supplementary Information Table 1^44^. As the creators of this database observe, this is by no means a comprehensive list of all possible human oral microbiome, immune response, and contaminant proteins, but rather provides the opportunity for a general assessment of archaeological samples by including the most commonly identified proteins from each group.

Total proteins identified by the OSSD were counted, as well as the number of proteins reflecting lab contaminants, common environmental contaminants, oral microbiome bacteria, and those produced as an immune response. Counts for each category are detailed in Table S1 and Fig. S1. The total number of microbiome protein IDs and the number of oral immune response proteins were combined, then this number was divided by the total number of identified OSSD proteins and then multiplied by 100 to determine a relative score of preservation. We assessed the preservation of the overall proteome of each calculus sample through the identification of PSMs in each sample for proteins included in the OSSD^28^. We set our preservation threshold at 45, which required that 45% of proteins be assigned to the oral microbiome or immune response. Thus, samples with a score above 45 and with at least ten total proteins are considered to have passed the assessment. Using the OSSD method with a suggested threshold of >45, we can assert that proteins normally found in the living human oral cavity make up almost half of the recovered OSSD proteins in the overall recovered sample proteome (Fig. S1). Those samples for which the expected oral proteins comprise at least 45% of the total OSSD proteins are considered to have a reasonably authentic “ancient” metaproteome. However, it is important to note that due to current technological limitations, there is, at present, no way to authenticate individual peptides; as we have outlined, authentication focuses on the entirety of the sample. The positive control (from archaeological sheep bone) and extraction blank (empty) for the sample batch are included here to show they did not contain any PSMs of oral microbiome bacteria or host immune response proteins.

### Archaeological information

Khorig I (MPI-SHH Department of Archeology codes: HOR-01, HOR-08, HOR-13, HOR-21, HOR-25, HOR-27, HOR-30, and HOR-40). Khorig II (MPI-SHH Department of Archeology codes: HOR-48, HOR-58, and HOR-73). See Tables S1, S3 for Laboratory IDs and original sample information, including burial number and year of excavation. These burial grounds are located in the northern forest-steppe region of Mongolia, in Khovsgol province^40,41^. The cemeteries are located at the top of two ridgelines in a forested area, with stone mounds dotted along the top of each ridge. The cemeteries are being actively looted; thus, our salvage operation attempted to recover and conserve as much material as possible. Each of the burials contained a single individual buried ~1.5 m below the surface. Burials were marked by a stone mound on the surface measuring ~0.5 m in height. As these were looted burials, human remains were recovered both from the surface and within burial pits. However, the majority of human remains and material culture (including ceramic vessels) were found during excavations of the bottom and sides of the burial chamber that remained undisturbed^42,43^. Samples were exported from Mongolia with Permit Number A/211 for the Mongolian National Museum granted on April 30, 2020. Samples were brought directly to the Max Planck Institute for the Science of Human History.

### Radiocarbon dating

Collagen was extracted from three human bones for radiocarbon dating at the Scottish Universities Environmental Research Centre (SUERC) Radiocarbon Dating Laboratory. Approximately 1 g of bone was mechanically cleaned of surface contaminant materials such as soil deposits. Collagen extraction followed a modified Longin (1971) protocol^65^. The clean bone was then demineralized in 100 mL of 1 M HCl over a period of at least 24 h until only a pseudomorph depleted of minerals could be observed. The acid solution was decanted and the pseudomorph was rinsed in ultrapure water. To solubilize the sample, 100 mL of ultrapure water was added and the sample container was placed in a sand bath, for an even heat distribution, at 80 °C for 3 h. The solution was then allowed to cool and was filtered using a GF/A paper filter (Whatman), after which it was dried using a freeze drier.

Dried collagen was combusted, graphitized, and AMS dated following a recent protocol^66^. The ^14^C/^13^C isotopic ratios of pressed graphite targets were measured using a National Electrostatics Corporation (NEC) 5MV tandem accelerator mass spectrometer. Sample runs included standard and background samples employed to correct for a background signal and to normalize the ^14^C/^13^C prior to age reporting. A fractionation correction relies on an offline measurement of the isotopic ratio of ^13^C and ^12^C in the analyzed sample, which is then compared to international standards NBS19 and IAEA-CO-1^66^. The preservation of bone collagen is assessed by measuring its carbon-to-nitrogen elemental atomic ratio (C/N) using a Costech ECS 4010 elemental analyzer (EA). Following previous work^67^, samples outside of the C/N range 2.9 to 3.6 are deemed unsuitable for reliable radiocarbon measurement.

### LC-MS/MS analysis

Mass spectrometry (LC-MS/MS) was conducted at the Functional Genomics Center Zurich using either a Q-Exactive or a Q-Exactive HF mass spectrometer (Thermo Scientific, Bremen, Germany) equipped with a Digital PicoView source (New Objective) and coupled to a nanoACQUITY or an ACQUITY UPLC M-Class system (Waters AG, Baden-Dättwil, Switzerland), respectively. Solvent composition at the two channels was 0.1% formic acid for channel A and 0.1% formic acid, 99.9% acetonitrile for channel B. Column temperature was 50 °C. For each sample, 4 μL of peptides were loaded on a commercial MZ Symmetry C18 Trap Column (100 Å, 5 µm, 180 µm × 20 mm, Waters) followed by nanoEase MZ C18 HSS T3 Column (100 Å, 1.8 µm, 75 µm × 250 mm, Waters). The peptides were eluted at a flow rate of 300 nL/min by a gradient from 8 to 22% B in 49 min, 32% B in 11 min, and 95% B in 1 min (Q-Exactive) or from 5 to 40% B in 120 min and 98% B in 5 min (Q-Exactive HF). The column was cleaned after each run with 98 % solvent B for 5 min and holding 98% B for 8 min prior to re-establishing the loading condition.

The mass spectrometers were operated in data-dependent mode performing HCD (higher-energy collision dissociation) fragmentation on the twelve most intense signals per cycle. The settings were slightly adapted for each instrument. For Q-Exactive analyses, full-scan MS spectra (300−1700 m/z) were acquired at a resolution of 70,000 at 200 m/z after accumulation to a target value (AGC) of 3E6, while HCD spectra were acquired at a resolution of 35,000 using a normalized collision energy of 25 (maximum injection time: 110 ms; AGC 50,000 ions). For Q-Exactive HF analyses, full-scan MS spectra (300−1500 m/z) were acquired at a resolution of 120,000 at 200 m/z after accumulation to a target value (AGC) of 3,000,000, while HCD spectra were acquired at a resolution of 30,000 using a normalized collision energy of 28 (maximum injection time: 50 ms; AGC 10,000 ions). Unassigned singly charged ions were excluded. Precursor masses previously selected for MS/MS measurement were excluded from further selection for 30 s, and the exclusion window was set at 10 ppm. The samples were acquired using internal lock mass calibration on m/z 371.1012 and 445.1200.

### Data analysis

To discern the best method for producing reliable results, we tested multiple settings in our database searches. First, we tested differences between known protein databases, running our samples against Swissprot and our custom dairy database. In addition, we ran all of our samples again against Swissprot, our custom dairy database, and the HOMD. For the most part, we found the same dietary proteins. Please see attached figure (Fig. S2), where all peptides identified from the original search (Swissprot, custom dairy database) and the HOMD search (Swissprot, custom dairy database, and HOMD) have been plotted.

While there could be false positives found in the initial searches, all initially identified peptides are further authenticated by comparing them against a database of all known protein sequences (well-annotated and those that are not annotated, even including automatic translations from genomic papers). We do this by using NCBI’s Protein-Protein BLAST alignment tool, found at: https://blast.ncbi.nlm.nih.gov/Blast.cgi. This allows us to be sure that identified peptides are unique to a specific species, genus, or family. BLAST searches are always done for each and every identified dietary protein.

Figure S2 indicates that for all dietary peptides, the species IDs and proteins are almost identical. In a few cases, our original search (blue) identified an extra peptide or two. In other cases, the HOMD search identified an extra peptide or two. We plotted them out to show the very small differences between these searches. Importantly, after searching against our original database with the HOMD, the species and protein IDs remain the same, with the only difference being the small shifts in the number of peptide spectral matches recovered. We explain how these differences happen in the paragraphs below.

When different databases are utilized, there are always slight variations between the results. This can happen for many reasons; however, the small changes between our original search and the original search databases + HOMD is due to the addition of protein sequences to the search space. Peptide MS/MS measurements are compared against the sequences in the database, and each peptide spectral match is scored in two ways: an ion score, which determines how likely it is that the amino acid sequence matches the particular species or tissue identification. In a sample, all peptide scores are relative to other peptides in the sample. The addition of a greater number of bacterial sequences into the database raises the total number of peptide IDs, which in turn alters the dynamic of the peptide scoring system. Occasionally, some peptides which met our scoring threshold for one database no longer meet that threshold against another database, and this is what occurred a few times between Swissprot + Dairy and SwissProt + Dairy + HOMD.

When there is a discrepancy between two database searches, it is not necessarily due to the peptide identifications no longer being reliable. In ancient samples, there are always modern contamination proteins that have been introduced to the overall proteome. These are often collagens and keratins from human handling, other bone materials within the burial or curation space, and bacteria from the soil environment. These introduced and modern proteins generally score much higher than the ancient proteins, as they are modern, they have not degraded as archaeological proteins have, and usually offer more complete protein coverage and are less affected by post-translational modifications. These high-scoring modern human and soil bacteria proteins can sometimes “swamp out” the relatively lower-scoring ancient proteins.

Secondly, we contemplated the use of tryptic versus semi-tryptic search settings for our samples. The use of semi-trypsin and trypsin settings on ancient samples has been explored in previous studies^34,68,69^. While using semi-trypsin is helpful when samples are very old (>10,000 years), the proteins contained within dental calculus do not appear to degrade much within time spans of less than 7000 years. However, looking at semi-tryptic results is something that we generally do with a few samples to see if there is a difference between the different settings.

One way to test for differences (between a tryptic and semi-tryptic search) is to look at the peptides recovered from a semi-tryptic search. The first step is to identify the precursor amino acids, or the amino acid that is just before the identified peptide, to see if the cleavage was made by trypsin or not. In our semi-tryptic searches, ~90% of the peptides had tryptic cuts. Furthermore, of the 10% that were non-tryptic, 86% of these were from collagen and keratin proteins listed as modern contaminants. There was only one identified non-tryptic peptide present within the proteins we would expect to be in the oral cavity (oral immune proteins and dietary proteins). Also, in the results of the semi-trypsin searches, all samples lost ~20% of protein and peptide IDs. However, the yak milk peptide remained present for each individual (HOR-27, HOR-01) in our study. The search parameters used for any study should always depend on the samples’ age and environment. In this case, with permafrost preservation and the young age of the samples, we are confident that fully tryptic searches are the proper choice.

### Reporting summary

Further information on research design is available in the Nature Portfolio Reporting Summary linked to this article.

## Supplementary information

Supplemental Material

Reporting Summary

## Data Availability

All raw, peak, and result from protein data has been uploaded to ProteomExchange (http://www.proteomexchange.org). Files are available under accession number: PXD024510 and project 10.6019/PXD024510.
