# Supplementary material for: Permafrost preservation reveals proteomic evidence for yak milk consumption in the 13th century
Source: Commun Biol. 2023 Mar 31;6:351. doi: 10.1038/s42003-023-04723-3 (PMC10066276; doi:10.1038/s42003-023-04723-3)
Supplement: Supplementary file 1 — Supplemental Material [file 42003_2023_4723_MOESM1_ESM.pdf]

Supplementary Information for

Permafrost preservation reveals proteomic evidence for yak milk consumption in 13th century

Alicia R. Ventresca Miller,[1,2,3]*† Shevan Wilkin,[3,4,5]*† Jamsranjav Bayarsaikhan,[3,6] Abigail Ramsøe,[7] Julia Clark,[8,9,10] Batsuren Byambadorj,[11] Sandra Vanderwarf,[8] Nils Vanwezer,[3] Ashleigh Haruda,[12,13] Ricardo Fernandes,[3,12,14] Bryan Miller,[2,3,15] Nicole Boivin[3,16,17,18]

Author Affiliations
[1] Department of Anthropology, University of Michigan, Ann Arbor 48109, Michigan, United States
[2] Museum of Anthropological Archaeology, University of Michigan, Ann Arbor 48109, Michigan, United States
[3] Department of Archaeology, Max Planck Institute for the Science of Human History, Kahlaische Strasse 10, 07745 Jena, Germany
[4] Institute for Evolutionary Medicine, Faculty of Medicine, University of Zürich, 8057 Zürich, Switzerland
[5] Australian Research Centre for Human Evolution (ARCHE), Griffith University, Brisbane 4111, Australia.
[6] National Museum of Mongolia, Juulchin Street-1, Ulaanbaatar, Mongolia
[7] Section for GeoGenetics, The GLOBE Institute, University of Copenhagen, Copenhagen, Denmark
[8] NOMAD Science, Glen, Montana
[9] Flinders University: Department of Archaeology, Flinders University, Bedford Park, 5042 Adelaide, SA, Australia
[10] Department of Sociology, Social Work and Anthropology, Utah State University, Logan, Utah
[11] Department of Anthropology and Archaeology, National University of Mongolia, Baga toiruu-44, Ulaanbaatar-46a, Mongolia
[12] School of Archaeology, University of Oxford, 1 South Parks Road, Oxford, UK.
[13] Department of Archaeology, University of Exeter, Laver Building, North Parks Road, Exeter, UK.
[14] Faculty of Arts, Masaryk University, Arne Nováka 1, 602 00 Brno-střed, Czechia
[15] History of Art Department University of Michigan, Ann Arbor 48109, Michigan, United States
[16] School of Social Science, University of Queensland, Brisbane, Australia
[17] Department of Archaeology, University of Calgary, Calgary, Canada
[18] Smithsonian Institution, New York, USA

Corresponding Author information
* Alicia R. Ventresca Miller, **Email:** avenmil@umich.edu
* Shevan Wilkin, **Email:** wilkin@shh.mpg.de
† These authors contributed equally to this work.

This PDF file includes:

Supplementary Note
Figures S1 to S4
Tables S1 to S4
SI References

Supplementary Note 1

Terminology

We have several terminological issues that we would like to address, these include discussions of *Bos mutus*/*grunniens*, cattle, and their hybrids [1], as well as our use of the terms BLG. As this paper is concerned with yak, we need to highlight that the peptide taxonomic assignment is to *Bos mutus* (wild yak) and that there are no domesticated yak (*Bos grunniens*) reference proteins in the NCBI database. However, a recent paper has detailed differences in the BLG and alpha lactalbumin sequences between *Bos taurus*, *Bos mutus*, *Bos grunniens*, and their hybrids [2]. Because of the dating of the samples in this study to the Medieval era (~1170-1390 CE), and the identification of dairying, it is very likely that the yak peptides we recovered were from domesticated yak (*Bos grunniens*) rather than wild yak (*Bos mutus*). Thus, in our manuscript we associate these peptides with domesticated yak or *Bos mutus*/*grunniens*. A similar issue is present with respect to our understanding of cattle, a term that broadly includes two species, *Bos taurus* and *Bos indicus*. Our use of the term cattle is for general readership, and similarly reflects the fact that the presence of cattle has been documented for this time-period and region. Finally, we use BLG as an acronym for β-Lactoglobulin, the whey protein of milk, rather than LGB the β-Lactoglobulin precursor. There are two isoforms of equine BLG, expressed by two genes LGB1 and LGB2. While LBG2 remains present in ruminants, it is no longer expressed. Thus, we have used the acronym BLG throughout the text when discussing all forms of β-Lactoglobulin.

Radiocarbon Dating

Three samples of human bone were sent for AMS radiocarbon dating to the SUERC Radiocarbon Laboratory in Glasgow. Their C/N atomic ratios were between 3.4 and 3.6 and thus the samples were considered suitable for radiocarbon dating (SUERC-96725: 3.4; SUERC-96726: 3.4; SUERC-96725: 3.6). AMS measurements following data reduction resulted in uncalibrated radiocarbon ages of 638 ± 29 BP (SUERC-96725), 698 ± 29 BP (SUERC-96726) and 826 ± 29 BP (SUERC-96727). Calibrated date ranges (95.4% C.I.) of samples HOR-01, HOR-08, and HOR-27 had the date following ranges: 1287-1397 CE, 1269-1388 CE, and 1170-1270 CE, respectively. Calibration was done using OxCal v. 4.4 software and the IntCal20 calibration curve for the northern hemisphere [3,4]. The difference between SUERC-96725 and SUERC-96727 (60 to 160 yrs for a 68.3 C.I. and 37 to 203 yrs for a 95.4% C.I.) provides an estimate on duration of use for the cemeteries from which the samples originate. While sample SUERC-96727 likely dates to the initial rise of Chinggis Khan and the Mongol Empire (1206-1271 CE), samples SUERC-96725 and SUERC- 96726 likely date to the later Yuan Dynasty period (1271-1368 CE).

Peptide Identifications by Individual

HOR-73, HOR-58, HOR-48 No dietary proteins were identified in the dental calculus of these individuals. Two of these samples passed initial screening HOR-73 (score of 71) and HOR-48 (score of 50), while HOR-58 failed to pass as it lacked immune response and oral microbiome proteins. During sampling, HOR-58 had little visible calculus and the total amount/weight of calculus analyzed was quite low (1 mg).

HOR-40 This individual's calculus passed screening with a score of 71 and contained numerous unique human salivary and immune proteins, as well as bacterial proteins common in the human oral microbiome. Additionally, there were two peptide spectral matches (PSMs) to BLG1 with species-specific identifications to *Equus*.

HOR-27 The calculus from this individual passed screening (score of 67) and contained PSMs for eight different milk proteins. Ruminant-specific proteins include BLG, beta-casein, alpha-S1-casein, kappa-casein, and alpha-S2-casein, and include identifications to Bovidae (cattle, sheep, and goat) and Bovinae (all *Bos* species), and importantly the first evidence for *Bos grunniens*/*mutus* (yak-specific) milk. The sample also contained PSMs for equine-specific milk proteins BLG1, Lysozyme C (milk isozyme), and alpha lactalbumin. Additionally, this individual also had one further dietary protein identified through PSMs for ruminant-specific hemoglobin subunit beta, which is a blood protein. This individual has a calibrated date range from 1170-1270 CE.

HOR-13 This individuals' calculus passed screening (score of 81) and contained PSMs for two horse milk proteins with matches to BLG1 and Lysozyme C (milk isozyme).

HOR-08 This individual's calculus has a preservation score of 79. Their calculus contained ruminant-specific peptide identifications for BLG and alpha-S1-casein, through PSMs that are taxonomically identified to Ovis, Caprinae (sheep or goat), and one taxonomically ambiguous peptide that can either be matched to sequences for Bovinae or Ovis. The sample additionally contained equine identifications to beta-casein and alpha-S2-casein, which represents the first identification of caseins from horse milk. The calibrated date range for this individual is from 1269 to 1388 CE.

HOR-30 With an oral signature preservation, score of 80 this individual did not have any peptide matches specific to milk proteins, but did contain three PSMs to *Equus* serum albumin, which is a horse protein found in tissues (for example blood, meat, fat, organs, sweat or urine).

HOR-01 This individual with a preservation score of 70 contains evidence for ruminant milk proteins BLG, kappa-casein, alpha-S1-casein, and alpha-S2-casein with taxonomic classifications for Pecora (all Artiodactyla), Bovidae, Bovinae, *Bos*, and the identification of *Bos grunniens*/*mutus* (yak). Furthermore, we identified two ruminant blood proteins, hemoglobin subunit alpha-1 and hemoglobin subunit beta. This individual has a calibrated date range from 1287 to 1397 CE.

HOR-21, HOR-25 The calculus samples from these two individuals passed the preservation score threshold (scores of 78 and 64 respectively) and contained PSMs for three different equine milk proteins including BLG1, Lysozyme C (milk isozyme), and alpha lactalbumin.

Fig. S1. Total proteins per sample based on OSSD results

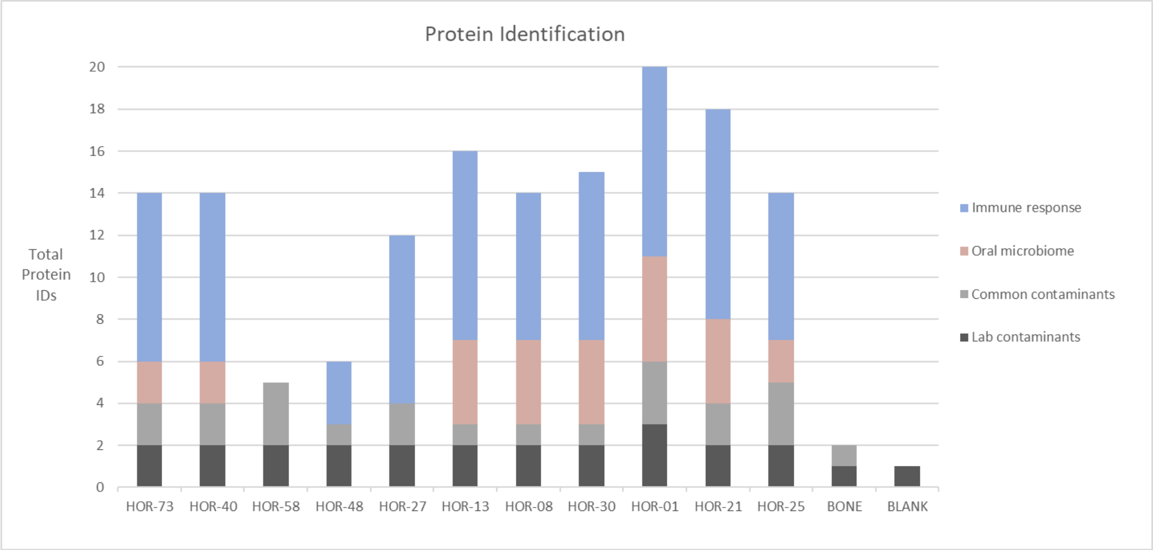

Fig. S2. Total counts of dietary PSMs identified through Swissprot and our custom dairy database (blue line) are compared to the count of dietary PSMs identified through Swissprot, our custom dairy database, and the Human Oral Microbiome Database (HOMD)(red line). Y-axis represents the number of PSMs recovered per database search.

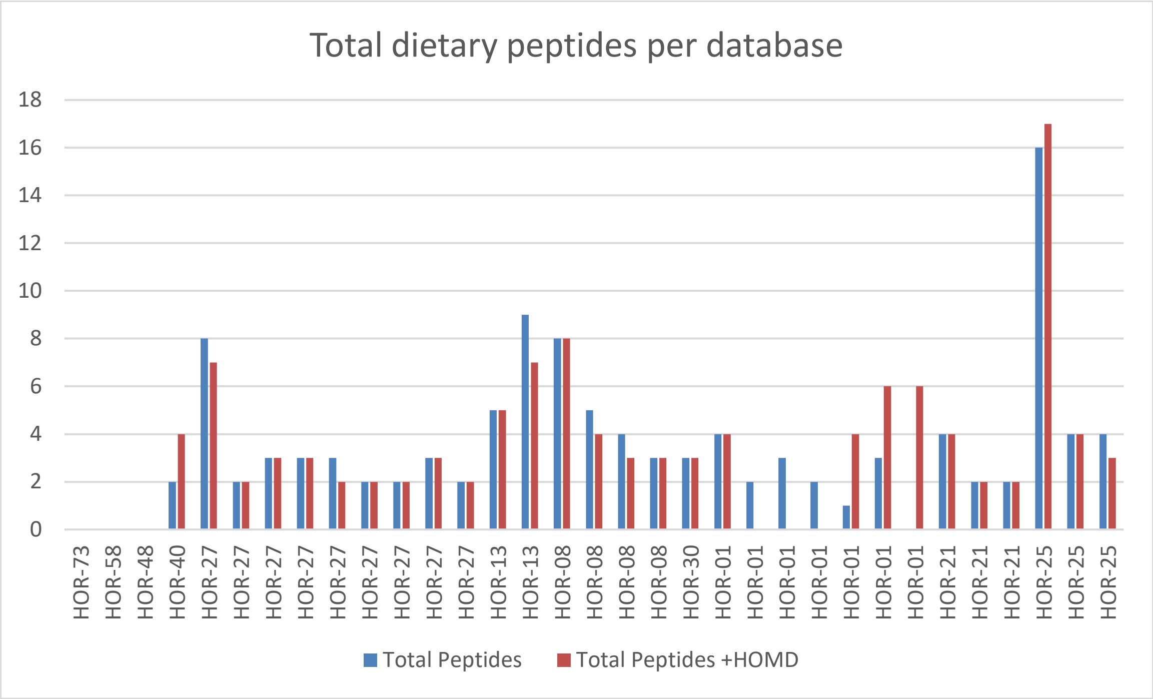

Fig. S3. Gold ornament in the form of a lotus encircling a seated Buddha that was recovered from the Khorig cemeteries

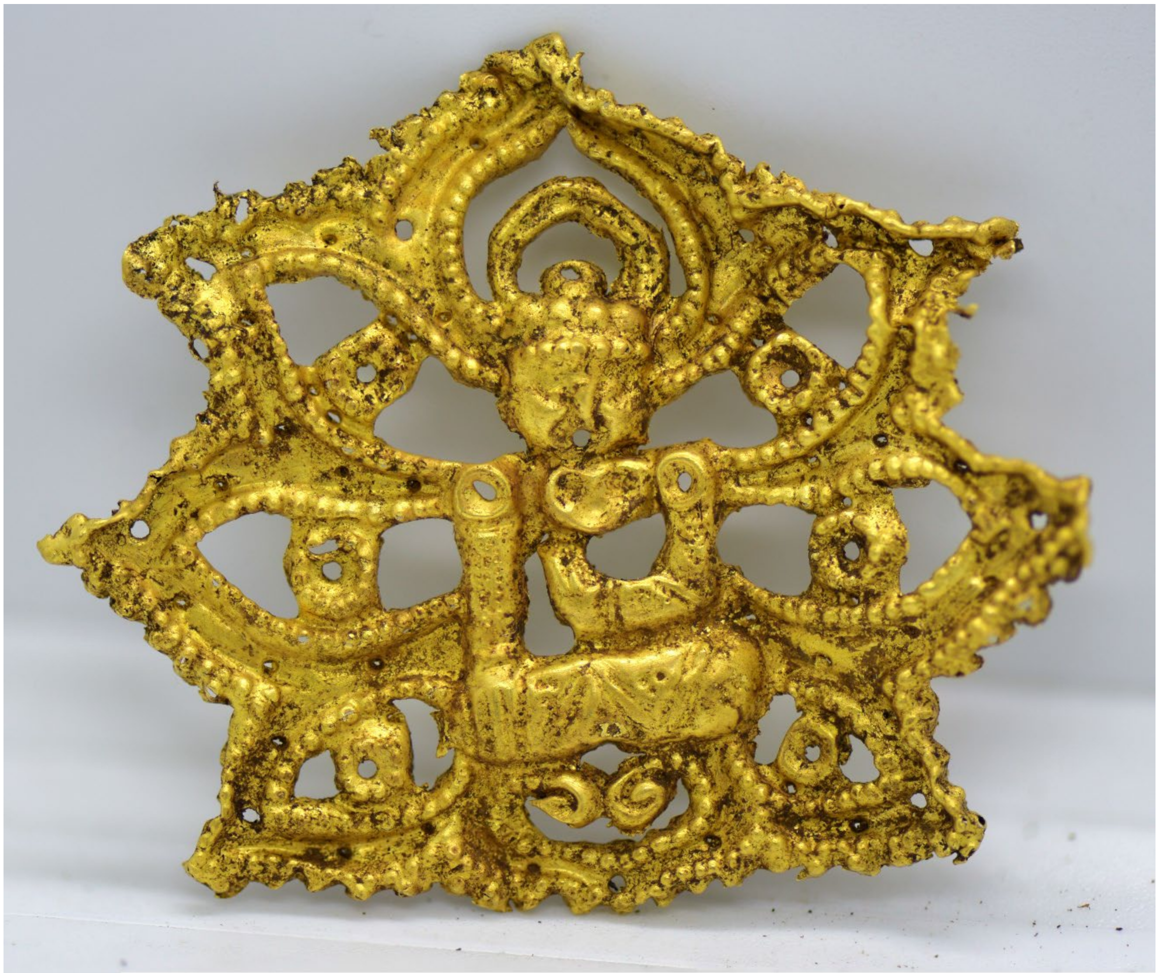

Fig. S4. Variety of petroglyph representations of *Bos* in Mongolia

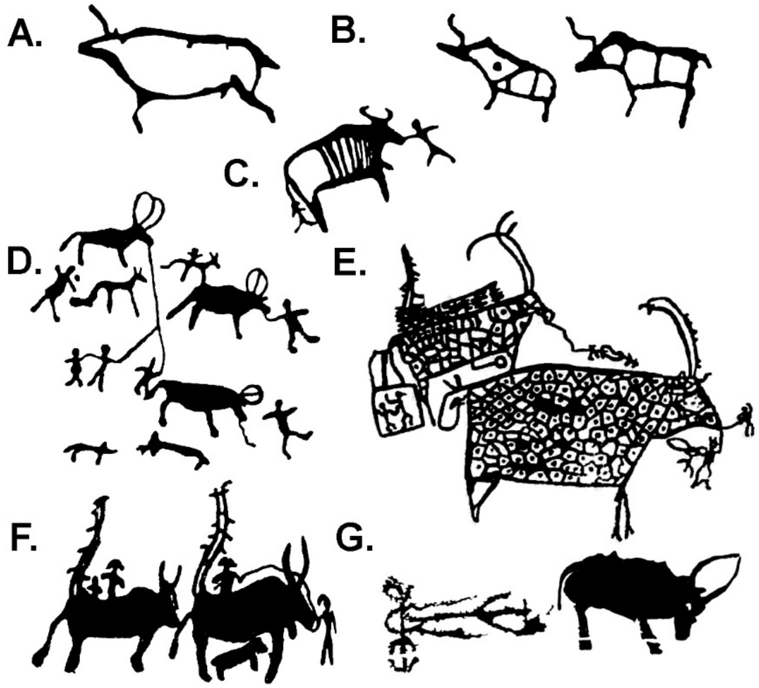

a Pre-Bronze Age aurochs denoted by forward facing horns. **b** Bronze Age aurochs. **c** Cattle held by two anthropomorphic figures. **d** Three cattle with leads held by several anthropomorphic figures, with horses and a horse rider, likely Late Bronze Age or later. **e** Two stylized *Bos* with leads held by anthropomorphic figures, the top one is carrying cargo and pulling a cart with two anthropomorphic figures. **f** Two *Bos* (likely yak) ridden by anthropomorphic figures and one with a lead/reins. **g** *Bos* (yak) pulling cart/chariot.

Table S1. Results of OSSD with total proteins identified, score (%), and whether the sample passed or failed the initial assessment.

Lab ID	Sample	Original Sample Information (Cemetery #, Burial #, year)	Dairy	Total protein IDS Mascot OSSD*	[illegible]s		Oral microbiome	Immune response	OSSD Score	PASS /FAIL
					Lab	Common				
DA892	HOR-73	Xorig II, L-7, 2019	No	14	2	2	2	8	71	PASS
DA894	HOR-40	Xorig I, L-26, 2019	Yes	14	2	2	2	8	71	PASS
DA895	HOR-58	Xorig II, L-4, 2019	No	5	2	3	0	0	0	FAIL
DA896	HOR-48	Xorig II, L-2 , 2019	No	6	2	1	0	3	50	PASS
DA897	HOR-27	Xorig I, L-1, 2019	Yes	12	2	2	0	8	67	PASS
DA898	HOR-13	Xorig I, L-15, 2019	Yes	16	2	1	4	9	81	PASS
DA899	HOR-08	Xorig I, L-14, 2019	Yes	14	2	1	4	7	79	PASS
DA900	HOR-30	NMAP, 350 , 2017	Yes	15	2	1	4	8	80	PASS
DA901	HOR-01	Xorig I, L12, 2019	Yes	20	3	3	5	9	70	PASS
DA902	HOR-21	Xorig I, L-17, 2019	Yes	18	2	2	4	10	78	PASS
DA903	HOR-25	Xorig I, L-7, 2019	Yes	14	2	3	2	7	64	PASS
DA912	BONE		No	2	1	1	0	0	0	-
DA914	BLANK		No	1	1	0	0	0	0	-

Table S2. Summary table of dietary proteins per individual.

Arch ID / Lab ID	Total Proteins°	Protein (PSMs)	Sequence	Mods	Peptide Taxonomic Assignment*	Peptide FDR	Protein FDR	E-Value Cutoff	Mascot Ion Score	Mascot E-value	Protein Taxonomic Assignment
HOR-73 /DA892	65	No dietary proteins	-	-	-	0.6	1.56	0.01			
HOR-58 /DA895	23	No dietary proteins	-	-	-	>0.01	>0.01	0.01			
HOR-48 /DA896	51	No dietary proteins	-	-	-	>0.01	>0.01	0.01			
HOR-40 /DA894	113	BLG I	VQIVPDLTR	NA	Equus	0.96	1.8	0.01	49.8	0.0049	Equus
			TQMVDEEIMEK	2 Oxidation (M)	Equus				28.08	0.0023	
HOR-27 /DA897	158	BLG I	ALQPLPGR	Deamidated (NQ)	Equus	0.53	1.94	0.05	30.42	0.042	Bos grunniens/ mutus, Bovidae, Bovinae, Equus
			TESPAEFK	NA	Equus				39.82	2.00E-02	
			VQIVPDLTR	NA	Equus				47.09	9.10E-03	
			VQIVPDLTR	Deamidated (NQ)	Equus				32.78	0.017	
			TQMVDEEIMEK	2 Oxidation (M)	Equus				33.56	0.00071	
			TQMVDEEIMEK	Deamidated (NQ); 2 Oxidation (M)	Equus				40.93	0.00015	
			LRPTPEDNLEIILR	NA	Equus				37.48	0.0088	
			NAATPGQSLVCQYLAR	NA	Equus				26.64	0.0032	
		Lysozyome C, milk isozyme	LLDENIDDDISCAK	NA	Equus				45.92	4.90E-05	
			NANGSSDYGLFQLNNK	Deamidated (NQ)	Equus				52.76	0.0057	
		Hemoglobin subunit beta	VDEVGAEALGR	NA	Pecora				42.33	0.002	
			LLVVYPWTQR	NA	Not unique				27.99	0.032	
			VKVDEVGAEALGR	NA	Pecora				52.35	0.0018	
		Beta-casein	VLPVPQK	NA	Not unique				18.19	0.029	
			DMPIQAFLLYQEPVLGPVR	Oxidation (M)	Bovidae				43.9	9.70E-05	
			DMPIQAFLLYQEPVLGPVR	Oxidation (M)	Bovidae				28.05	0.0029	
		Alpla lactalbumin	DNQILPSR	NA	Equus				44.55	7.40E-04	
			FLDDDLTDDVMCAK	Oxidation (M)	Equus				28.3	0.0022	
			NNGKTEYGLFQINNK	3 Deamidated (NQ)	Not unique				16.01	0.031	
		Alpha-S1-casein	YLGYLEQLLR	NA	Not unique				45.49	2.60E-04	
			FFVAPFPEVFGK	NA	Bovinae				19.13	0.016	
		Kappa casein	YIPIQYVLSR	NA	Pecora				26.32	0.017	
			SPAQILQWQVLSNTVPAK	NA	Bovinae				36.69	3.60E-04	
		Alpha-S2-casein	TKVIPYVR	NA	Not unique				26.19	0.021	
			ITVDDKHYQK	NA	Bovidae				25.71	0.039	
			ALNEINQFYQK	NA	Bovidae				19.67	0.016	
		BLG	VLVLDTDYKK	NA	Pecora				25.8	0.0038	
			LSFNPTQLEGQCHI	NA	Bos mutus				30.97	0.0012	

Arch ID / Lab ID	Total Proteins°	Protein (PSMs)	Sequence	Mods	Peptide Taxonomic Assignment*	Peptide FDR	Protein FDR	E-Value Cutoff	Mascot Ion Score	Mascot E-value	Protein Taxonomic Assignment
HOR-73 /DA892	65	No dietary proteins	-	-	-	0.6	1.56	0.01			
HOR-58 /DA895	23	No dietary proteins	-	-	-	>0.01	>0.01	0.01			
HOR-48 /DA896	51	No dietary proteins	-	-	-	>0.01	>0.01	0.01			
HOR-40 /DA894	113	BLG I	VQIVPDLTR	NA	Equus	0.96	1.8	0.01	49.8	0.0049	Equus
			TQMVDEEIMEK	2 Oxidation (M)	Equus				28.08	0.0023	
HOR-27 /DA897	158	BLG I	ALQPLPGR	Deamidated (NQ)	Equus	0.53	1.94	0.05	30.42	0.042	Bos grunniens/ mutus, Bovidae, Bovinae, Equus
			TESPAEFK	NA	Equus				39.82	2.00E-02	
			VQIVPDLTR	NA	Equus				47.09	9.10E-03	
			VQIVPDLTR	Deamidated (NQ)	Equus				32.78	0.017	
			TQMVDEEIMEK	2 Oxidation (M)	Equus				33.56	0.00071	
			TQMVDEEIMEK	Deamidated (NQ); 2 Oxidation (M)	Equus				40.93	0.00015	
			LRPTPEDNLEIILR	NA	Equus				37.48	0.0088	
			NAATPGQSLVCQYLAR	NA	Equus				26.64	0.0032	
		Lysozyome C, milk isozyme	LLDENIDDDISCAK	NA	Equus				45.92	4.90E-05	
			NANGSSDYGLFQLNNK	Deamidated (NQ)	Equus				52.76	0.0057	
		Hemoglobin subunit beta	VDEVGAEALGR	NA	Pecora				42.33	0.002	
			LLVVYPWTQR	NA	Not unique				27.99	0.032	
			VKVDEVGAEALGR	NA	Pecora				52.35	0.0018	
		Beta-casein	VLPVPQK	NA	Not unique				18.19	0.029	
			DMPIQAFLLYQEPVLGPVR	Oxidation (M)	Bovidae				43.9	9.70E-05	
			DMPIQAFLLYQEPVLGPVR	Oxidation (M)	Bovidae				28.05	0.0029	
		Alpla lactalbumin	DNQILPSR	NA	Equus				44.55	7.40E-04	
			FLDDDLTDDVMCAK	Oxidation (M)	Equus				28.3	0.0022	
			NNGKTEYGLFQINNK	3 Deamidated (NQ)	Not unique				16.01	0.031	
		Alpha-S1-casein	YLGYLEQLLR	NA	Not unique				45.49	2.60E-04	
			FFVAPFPEVFGK	NA	Bovinae				19.13	0.016	
		Kappa casein	YIPIQYVLSR	NA	Pecora				26.32	0.017	
			SPAQILQWQVLSNTVPAK	NA	Bovinae				36.69	3.60E-04	
		Alpha-S2-casein	TKVIPYVR	NA	Not unique				26.19	0.021	
			ITVDDKHYQK	NA	Bovidae				25.71	0.039	
			ALNEINQFYQK	NA	Bovidae				19.67	0.016	
		BLG	VLVLDTDYKK	NA	Pecora				25.8	0.0038	
			LSFNPTQLEGQCHI	NA	Bos mutus				30.97	0.0012	

Arch ID / Lab ID	Total Proteins°	Protein (PSMs)	Sequence	Mods	Peptide Taxonomic Assignment*	Peptide FDR	Protein FDR	E-Value Cutoff	Mascot Ion Score	Mascot E-value	Protein Taxonomic Assignment
HOR-13 /DA898	183	Lysozyme C, milk isozyme	KVFSKCELAHK	NA	Equus	0.45	1.1	0.05	26.67	0.0087	Equus
			LLDENIDDDISCAK	NA	Equus				54.99	7.00E-06	
			LLDENIDDDISCAK	NA	Equus				43.82	7.80E-05	
			LLDENIDDDISCAKR	NA	Equus				35.13	5.10E-04	
			LLDENIDDDISCAKR	NA	Equus				29.97	0.0015	
		BLG I	VQIVPDLTR	NA	Equus				37.12	0.013	
			VQIVPDLTR	NA	Equus				49.56	0.0052	
			TQMVDEEIMEK	2 Oxidation (M)	Equus				30.67	0.0013	
			LRPTPEDNLEIILR	NA	Equus				51.03	0.0081	
			LRPTPEDNLEIILR	NA	Equus				16.72	0.034	
			LRPTPEDNLEIILR	NA	Equus				46.79	0.011	
			TQMVDEEIMEKFR	2 Oxidation (M)	Equus				17.76	0.022	
			NAATPGQSLVCQYLAR	NA	Equus				23.18	0.0067	
			NAATPGQSLVCQYLAR	Deamidated (NQ)	Equus				15.89	0.034	
HOR-08 /DA899	334	BLG	TKIPAVFK	NA	Not unique	1.18	2.14	0.05	49.69	0.0037	Ovis, Caprinae, Bovinae/Ovis, Equus
			IIVTQTMK	Oxidation (M)	Not unique				42.8	0.0019	
			VLVLDTDYKK	NA	Pecora				50.1	2.00E-05	
			TPEVDNEALEK	NA	Ovis				44.73	0.0031	
			TPEVDNEALEKFDK	Deamidated (NQ)	Bovinae/Ovis				23.48	0.0063	
			TPEVDNEALEKFDK	Deamidated (NQ)	Bovinae/Ovis				15.66	0.034	
			TPEVDNEALEKFDK	Deamidated (NQ)	Bovinae/Ovis				51.17	0.011	
			IDALNENKVLVLDTDYKK	Deamidated (NQ)	Pecora				27.43	0.03	
		Alpha S1 casein	YLGYLEQLLR	NA	Bovidae				33.26	0.0032	
			FVVAPFPEVFR	NA	Caprinae				43.71	3.50E-04	
			YIQKEDVPSER	NA	Caprinae				31.89	0.024	
			YIQKEDVPSER	Deamidated (NQ)	Caprinae				25.81	0.013	
			FVVAPFPEVFRK	NA	Caprinae				22.71	0.0074	
		Beta casein	QILNPTNGENLR	NA	Equus (and also tapir)				20.07	0.013	
			QILNPTNGENLR	Deamidated (NQ)	Equus (and also tapir)				36.39	4.60E-04	
			VAPFPQPVVPYPQR	NA	Equus				18.35	0.019	
			DTPVQAFLLYQDPR	NA	Equus				39.44	2.20E-04	
		Alpha S2 casein	FTLPQYFK	NA	Equus				38.17	0.0047	
			TNSYQIIPVLR	NA	Equus				38.28	7.00E-04	
			TELTEEEKNYLK	NA	Equus				28.51	0.0036	
HOR-30 DA900	131	Serum albumin	TVLGNFSAFVAK	NA	Equus	0.81	1.55	0.01	47.36	1.10E-04	Equus
			RHPDYSVSLLLR	NA	Equus				48.28	1.60E-04	
			KAPQVSTPTLVEIGR	NA	Equus				51.64	1.20E-04	

Arch ID / Lab ID	Total Proteins°	Protein (PSMs)	Sequence	Mods	Peptide Taxonomic Assignment*	Peptide FDR	Protein FDR	E-Value Cutoff	Mascot Ion Score	Mascot E-value	Protein Taxonomic Assignment
HOR-01 /DA901	206	BLG	VLVLDTDYKK	NA	Pecora	0.91	3	0.05	39.51	2.00E-04	Bos grunniens/ mutus, Bos, Bovinae, Pecora
			TPEVDDEALEKFDK	NA	Bovinae/Ovis				38.47	2.50E-04	
			LSFNPTQLEGQCHI	NA	Bos mutus				21.6	0.022	
			IDALNENKVLVLDTDYKK	NA	Pecora				31.51	0.0011	
		Kappa casein	YIPIQYVLSR	NA	Pecora				33.79	0.0048	
			SPAQILQWQVLSNTVPAK	NA	Bovinae				56.49	5.00E-06	
		Alpha S1 casein	YLGYLEQLLR	NA	Bovidae				26.61	0.012	
			HQGLPQEVLNENLLR	NA	Pecora				26.57	0.025	
			YKVPQLEIVPNSAEER	NA	Bovinae (likely Bos)				14.75	0.041	
		Alpha S2 casein	TKVIPYVR	NA	Bovinae				18.83	0.042	
			NMAINPSKENLCSTFCK	Oxidation (M)	Bos				19.84	0.014	
		Beta casein	VLPVPQK	NA	Not unique				17.58	0.027	
		Hemoglobin subunit alpha-1	VGGNAGAYGAEALER	NA	Bovidae				39.25	0.0027	
		Serum albumin	HLVDEPQNLIKK	NA	Caprinae				31.63	0.025	
			HGEYGFQNALIVR	NA	Caprinae (and Tibetan antelope)				36.54	0.033	
			HGEYGFQNALIVR	NA	Caprinae (and Tibetan antelope)				17.69	0.036	
			KAPQVSTPTLVEISR	NA	Not unique				28.95	0.011	
			KAPQVSTPTLVEISR	NA	Not unique				37.18	0.0076	
			RPCFSDLTLDETYVPKPFDEK	NA	Ovis aries				19.03	0.029	
		Hemoglobin subunit beta	AAVTGFWGK	NA	Pecora				25.67	0.044	
			VKVDEVGAEALGR	NA	Pecora				49.21	0.0049	
			VKVDEVGAEALGR	NA	Pecora				36.48	9.80E-04	
HOR-21 /DA902	173	BLG I	VQIVPDLTR	NA	Equus	1.05	2.37	0.05	49.58	0.0052	Equus
			TQMVDEEIMEK	2 Oxidation (M)	Equus				57.34	1.60E-05	
			LRPTPEDNLEIILR	NA	Equus				32.43	0.022	
			NAATPGQSLVCQYLAR	NA	Equus				58.81	3.10E-06	
		Lysozyme C, milk isozyme	LLDENIDDDISCAK	NA	Equus				50.65	1.80E-05	
			LLDENIDDDISCAKR	NA	Equus				27.08	0.008	
		Alpha lactalbumin	DNQILPSR	NA	Equus				34.6	0.009	
			FLDDDLTDDVMCAK	Oxidation (M)	Equus				37.58	5.10E-04	
HOR-25 /DA903	134	BLG I	VQIVPDLTR	NA	Equus	0.38	0.75	0.01	47.08	0.0093	Equus
			TQMVDEEIMEK	Oxidation (M)	Equus				41.98	2.90E-04	
			TQMVDEEIMEK	2 Oxidation (M)	Equus				30.08	0.0015	
			TQMVDEEIMEK	2 Oxidation (M)	Equus				49.83	3.10E-05	
			TQMVDEEIMEK	2 Oxidation (M)	Equus				37.65	2.90E-04	
			TQMVDEEIMEK	Deamidated (NQ);	Equus				27.82	0.0025	
			IFAEKTESPAEFK	NA	Equus				26.63	0.0032	
			LRPTPEDNLEIILR	NA	Equus				50.1	0.0099	
			LRPTPEDNLEIILR	NA	Equus				33.52	0.0033	
			LRPTPEDNLEIILR	NA	Equus				50.91	0.0055	
			LRPTPEDNLEIILR	Deamidated (NQ)	Equus				50.79	0.0011	
			LRPTPEDNLEIILR	Deamidated (NQ)	Equus				53.87	0.0046	
			LRPTPEDNLEIILR	Deamidated (NQ)	Equus				46.89	0.0017	
			NAATPGQSLVCQYLAR	NA	Equus				58.7	3.10E-06	
			NAATPGQSLVCQYLAR	Deamidated (NQ)	Equus				47.13	3.80E-05	
			NAATPGQSLVCQYLAR	Deamidated (NQ)	Equus				31.83	0.001	
		Lysozyme C, milk isozyme	LLDENIDDDISCAK	NA	Equus				66.87	5.40E-07	
			LLDENIDDDISCAK	Deamidated (NQ)	Equus				38.58	2.40E-04	
			LLDENIDDDISCAKR	NA	Equus				37.87	2.80E-04	
			LLDENIDDDISCAKR	NA	Equus				33.97	6.50E-04	
		Alpha lactalbumin	DNQILPSR	NA	Equus				47.92	6.80E-04	
			NICGISCNK	NA	Equus				35.14	0.0019	
			FLDDDLTDDVMCAK	Oxidation (M)	Equus				61.98	2.20E-06	

Table S3. Summary table of radiocarbon dates.

Arch ID	Lab ID	Original Sample Information (Cemetery #, Burial #, year)	Radiocarbon Laboratory Numbers	Uncal. Radiocarbon Dates	Cal. Radiocarbon Dates	Protein Taxonomic Assignments (identified in associated dental calculus samples)
HOR-73	DA892	Xorig II, L-7, 2019	Undated	Undated	Undated	No dietary proteins
HOR-58	DA895	Xorig II, L-4, 2019	Undated	Undated	Undated	No dietary proteins
HOR-48	DA896	Xorig II, L-2 , 2019	Undated	Undated	Undated	No dietary proteins
HOR-40	DA894	Xorig I, L-26, 2019	Undated	Undated	Undated	Equus
HOR-27	DA897	Xorig I, L-1, 2019	SUERC-96727	826 ± 29 BP	1170-1270	Bos grunniens/mutus, Bovidae, Bovinae,
HOR-13	DA898	Xorig I, L-15, 2019	Undated	Undated	Undated	Equus
HOR-08	DA899	Xorig I, L-14, 2019	SUERC-96726	698 ± 29 BP	1269-1388	Ovis, Caprinae, Bovinae/Ovis, Equus
HOR-30	DA900	NMAP, 350 , 2017	Undated	Undated	Undated	Equus
HOR-01	DA901	Xorig I, L12, 2019	SUERC-96725	638 ± 29 BP	1287-1397	Bos grunniens/mutus, Bos, Bovinae,
HOR-21	DA902	Xorig I, L-17, 2019	Undated	Undated	Undated	Equus
HOR-25	DA903	Xorig I, L-7, 2019	Undated	Undated	Undated	Equus

Table S4. Summary table linking individuals, laboratory IDs, and data files.

Individual	Lab ID	Raw File	MGF	MZID
HOR-73	DA892	DA892_F007388.raw	F007388.mgf	DA892_F007388.mzid.gz
HOR-40	DA894	DA894_F007390.raw	F007390.mgf	DA894_F007390.mzid.gz
HOR-58	DA895	DA895_F007391.raw	F007391.mgf	DA895_F007391.mzid.gz
HOR-48	DA896	DA896_F007392.raw	F007392.mgf	DA896_F007392.mzid.gz
HOR-27	DA897	DA897_F007393.raw	F007393.mgf	DA897_F007393.mzid.gz
HOR-13	DA898	DA898_F007394.raw	F007394.mgf	DA898_F007394.mzid.gz
HOR-08	DA899	DA899_F007395.raw	F007395.mgf	DA899_F007395.mzid.gz
HOR-30	DA900	DA900_F007396.raw	F007396.mgf	DA900_F007396.mzid.gz
HOR-01	DA901	DA901_F007397.raw	F007397.mgf	DA901_F007397.mzid.gz
HOR-21	DA902	DA902_F007398.raw	F007398.mgf	DA902_F007398.mzid.gz
HOR-25	DA903	DA903_F007399.raw	F007399.mgf	DA903_F007399.mzid.gz
Pos Control	DA912	DA912_F007407.raw	F007407.mgf	DA912_F007407.mzid.gz
Blank	DA914	DA914_F007409.raw	F007409.mgf	DA914_F007409.mzid.gz

SI References

1. Medugorac, I. *et al.* Whole-genome analysis of introgressive hybridization and characterization of the bovine legacy of Mongolian yaks. *Nat Genet* **49**, 470–475 (2017).
2. Wang, L., Ma, Y., Li, H., Yang, F. & Cheng, J. Identification and characterization of yak α-lactalbumin and β-lactoglobulin. *Journal of Dairy Science* **104**, 2520–2528 (2021).
3. Bronk Ramsey, C. Bayesian Analysis of Radiocarbon Dates. *Radiocarbon* **51**, 337–360 (2009).
4. Reimer, P. J. Composition and consequences of the IntCal20 radiocarbon calibration curve. *Quat. res.* **96**, 22–27 (2020).
