# Supplementary material for: Permafrost preservation reveals proteomic evidence for yak milk consumption in the 13th century
Source: Commun Biol. 2023 Mar 31;6:351. doi: 10.1038/s42003-023-04723-3 (PMC10066276; doi:10.1038/s42003-023-04723-3)
Supplement: Supplementary file 2 — Reporting Summary [file 42003_2023_4723_MOESM2_ESM.pdf]

nature portfolio

Corresponding author(s): Double-blind peer review submissions: write DBPR and your manuscript number here instead of author names.

Last updated by author(s): YYYY-MM-DD

Reporting Summary

Nature Portfolio wishes to improve the reproducibility of the work that we publish. This form provides structure for consistency and transparency in reporting. For further information on Nature Portfolio policies, see our Editorial Policies and the Editorial Policy Checklist.

Statistics

For all statistical analyses, confirm that the following items are present in the figure legend, table legend, main text, or Methods section.

n/a	Confirmed	
☒	☐	The exact sample size (*n*) for each experimental group/condition, given as a discrete number and unit of measurement
☐	☒	A statement on whether measurements were taken from distinct samples or whether the same sample was measured repeatedly
☒	☐	The statistical test(s) used AND whether they are one- or two-sided<br>*Only common tests should be described solely by name; describe more complex techniques in the Methods section.*
☒	☐	A description of all covariates tested
☒	☐	A description of any assumptions or corrections, such as tests of normality and adjustment for multiple comparisons
☒	☐	A full description of the statistical parameters including central tendency (e.g. means) or other basic estimates (e.g. regression coefficient) AND variation (e.g. standard deviation) or associated estimates of uncertainty (e.g. confidence intervals)
☒	☐	For null hypothesis testing, the test statistic (e.g. *F*, *t*, *r*) with confidence intervals, effect sizes, degrees of freedom and *P* value noted<br>*Give P values as exact values whenever suitable.*
☒	☐	For Bayesian analysis, information on the choice of priors and Markov chain Monte Carlo settings
☒	☐	For hierarchical and complex designs, identification of the appropriate level for tests and full reporting of outcomes
☒	☐	Estimates of effect sizes (e.g. Cohen's *d*, Pearson's *r*), indicating how they were calculated

Our web collection on statistics for biologists contains articles on many of the points above.

Software and code

Policy information about availability of computer code

Data collection	*Provide a description of all commercial, open source and custom code used to collect the data in this study, specifying the version used OR state that no software was used.*
Data analysis	PSM identifications via Mascot were filtered using an in-house developed tool MS-MARGE (64; freely available at https://bitbucket.org/rwhagan/ms-marge/src/master/).<br>Resulting raw MS/MS data were converted to Mascot generic files (mgf) using MSCovert from ProteoWizard (v.3.0.11781) (62). These files were then searched against Swissprot and a custom curated dairy protein database (30) using Mascot MS/MS ion search engine (v.2.6.0) (63).

For manuscripts utilizing custom algorithms or software that are central to the research but not yet described in published literature, software must be made available to editors and reviewers. We strongly encourage code deposition in a community repository (e.g. GitHub). See the Nature Portfolio guidelines for submitting code & software for further information.

Data

Policy information about availability of data

All manuscripts must include a data availability statement. This statement should provide the following information, where applicable:

- Accession codes, unique identifiers, or web links for publicly available datasets
- A description of any restrictions on data availability
- For clinical datasets or third party data, please ensure that the statement adheres to our policy

Provide your data availability statement here.

Human research participants

Policy information about studies involving human research participants and Sex and Gender in Research.

Reporting on sex and gender	N/A
Population characteristics	N/A
Recruitment	N/A
Ethics oversight	N/A

Note that full information on the approval of the study protocol must also be provided in the manuscript.

Field-specific reporting

Please select the one below that is the best fit for your research. If you are not sure, read the appropriate sections before making your selection.

☐ Life sciences ☐ Behavioural & social sciences ☒ Ecological, evolutionary & environmental sciences

For a reference copy of the document with all sections, see nature.com/documents/nr-reporting-summary-flat.pdf

Ecological, evolutionary & environmental sciences study design

All studies must disclose on these points even when the disclosure is negative.

Study description	To examine the potential of paleoproteomics to shed light on domesticated yak in Mongolia, we analyzed human dental calculus from Mongol era elite individuals recovered from permafrost burials in Khovsgol province, where people continue to herd yak to this day.
Research sample	Proteins were extracted from the dental calculus of 11 individuals buried in a Mongol era cemetery. The cemetery was excavated as part of a salvage excavation in northern Mongolia.
Sampling strategy	Every individual that was available was sampled.
Data collection	Data was collected from each individual that had dental calculus.
Timing and spatial scale	All samples were collected during the summer of 2019, from burials that were excavated in 2018 and 2019.
Data exclusions	All data were included in the analysis. Individuals lacking ancient proteins in dental calculus were discussed.
Reproducibility	The process of extraction of proteins is well developed. However, dental calculus from buried contexts is a finite resource. The remaining extracts could be re-analyzed from a portion of the samples.
Randomization	N/A
Blinding	Blinding was not relevant to this study, which reports the identification of ancient proteins in dental calculus. However, we did use a positive control and a blank during extraction of proteins.

Did the study involve field work? ☒ Yes ☐ No

Field work, collection and transport

Field conditions	Fieldwork occurred in northern Mongolia. The conditions were cold and rainy.
Location	Samples were collected during analysis of human remains. They were collected inside of a ger/yurt.
Access & import/export	All materials were imported into Germany following standard protocols. We have the necessary excavation permits and import permits.
Disturbance	Excavations were of looted burials, thus our work helped to minimize the damage that was done. All burials were refilled after our excavations.

Reporting for specific materials, systems and methods

We require information from authors about some types of materials, experimental systems and methods used in many studies. Here, indicate whether each material, system or method listed is relevant to your study. If you are not sure if a list item applies to your research, read the appropriate section before selecting a response.

Materials & experimental systems

n/a	Involved in the study	
☒	☐	Antibodies
☒	☐	Eukaryotic cell lines
☐	☒	Palaeontology and archaeology
☒	☐	Animals and other organisms
☒	☐	Clinical data
☒	☐	Dual use research of concern

Methods

n/a	Involved in the study	
☒	☐	ChIP-seq
☒	☐	Flow cytometry
☒	☐	MRI-based neuroimaging

Palaeontology and Archaeology

Specimen provenance	A certificate of origin (Reference No MN DE 7 14583) was provided from the National Museum of Mongolia for export of samples to Dr. Alicia Ventresca Miller (MPI-SHH). This was done through the Mongolian national chamber of commerce and industry. We also have excavation permits from 2018 and 2019.
Specimen deposition	The dental calculus has been fully destroyed from our analyses. If any extracts exist they are housed in the MPI-SHH palaeoproteomics laboratory.
Dating methods	New dates were provided from bone. All information is in the manuscript, regarding raw and calibrated dates.

☒ Tick this box to confirm that the raw and calibrated dates are available in the paper or in Supplementary Information.

Ethics oversight	The National Museum of Mongolia provided ethical oversight of this project.

Note that full information on the approval of the study protocol must also be provided in the manuscript.
